# Liverpool and Tropical Diseases

**Published:** 1903-04-11

**Authors:** 


### Liverpool and Tropical Diseases.

Few things could better illustrate the serious character of the friendly rivalry which, during the last few years, has sprung into existence as between the medical schools of London and of the great provincial cities than the report for the year 1902, just issued, of the Liverpool School of Tropical Medicine. It is but the fourth report, the school being of quite recent origin, but it describes an amount of achievement, alike in Liverpool in the work of teaching, and in other parts of the world in the direction of carrying the teaching into practice, of which everyone concerned may be justly proud. The special ward in the Royal Southern Hospital, set apart for the business of the school, has received during the year 135 cases invalided from the tropics, and including a great variety of nationalities, as well as of the diseases which are the special subjects of study. Among the nationalities we find 74 English, 3 Scotch, 15 Irish, 7 Welsh, 3 Manx, and 10 East Indians; while Germans, French, Swedes, Dutch, Americans, Africans, Finns, Portuguese, Danes, and West Indians are represented by smaller numbers. Among the diseases were malaria and its consequences in various forms, blackwater fever, bilharzia, dysentery, beriberi, and hepatic abscess; while the general results of the treatment may fairly be described as satisfactory. Among the 135 cases there were only 10 deaths, two of them immediately after admission; while 81 cases were discharged "well" and 37 "relieved." The report informs us that the cases have been carefully studied, and that important methods for detecting the parasites of malaria, and of other diseases, have been elaborated by the staff from the material thus placed at their disposal; while four nurses have been specially trained at the expense of the school. The committee naturally point with much satisfaction to the distinctions which have been gained by members of the school during the year. The chairman, Sir Alfred Jones, has been made a Knight Commander of the Order of St. Michael and St. George; Professor Major Ronald Ross has been made a Companion of the Bath; and Professor Rupert Boyce has been elected a Fellow of the Royal Society. In addition to this, the Nobel prize for medicine for 1902 was awarded to Major Ronald Ross, in recognition of his valuable researches into malarial fever and tropical medicine, and was presented to him by his Majesty the King of Sweden on December 10th. The value of the prize was about £8,000. Major Ronald Ross has also been appointed to the Chair of Tropical Medicine at University College, founded in honour of Sir Alfred Jones, the originator and chairman of the school.

In addition to the ordinary business of teaching and of investigation in Liverpool, the school has sent out four new expeditions during the year. The first of these was to Sierra Leone, under Major Ronald Ross, for the purpose of ascertaining the results of the operations conducted by Dr. Logan Taylor, at Freetown, for the destruction of the anopheles. Major Ross reported that the campaign was being carried on by Dr. Taylor with energy and tact, and that about 70 men were being employed at the expense of the school for the purpose of draining the mosquito-breeding pools, and removing the rubbish, such as old tins and broken bottles, which had remained in the backyards of the houses of the town for years and in which innumerable mosquitoes had been breeding. It was impossible to obtain precise evidence as to the effects of these measures on the health of the population, because there were no trustworthy statistics of former periods to be used for the purposes of comparison, but there was a marked reduction in the number of mosquitoes, and, according to the general statements of the residents, the health of the town was remarkably good and there had been only a few deaths during the year. A second expedition, under the direction of Major Ross, was sent to Ismailia at the request of the Suez Canal Company, as communicated in a letter from its president, Prince d'Arenberg, to investigate the causes of the prevalence there of malaria, and to recommend measures for its prevention. Major Ross was joined by Sir William MacGregor, K.C.M.G. A third expedition, in charge of Dr. Dutton and Dr. J. L. Todd, has been despatched to the Gambia and French Senegal, chiefly for the study of trypanosomiasis. A fourth, under Dr. Logan Taylor, has proceeded to Cape Coast Castle, in consequence of the unfavourable reports which had been received as to the health of the Europeans in the Gold Coast Colony. Negotiations are in progress between the school and the authorities of the Congo Free State, with regard to the despatch of experts to the Congo to investigate and report on the prevalence there of certain maladies. Dr. Dutton and Dr. Todd will therefore be despatched to Leopoldville on the termination of their work in the Gambia and Senegal. Altogether, the record is an eminently distinguished one, and is not only highly creditable to the enterprise of the great city from which it proceeds, but also displays this country in her rightful position as a leader in everything which tends to contribute to the health and longevity of the human race. The great problems of beriberi and of sleeping sickness still await solution in the localities where they respectively prevail, as in parts of India and in Uganda; and, with respect to these, it may be expected that the London school will soon show itself to have been no whit behind its provincial rivals either in enterprise, or in the power of bringing difficult undertakings to a successful issue.

